# Testing of a Dual Process Model to Resolve the Socioeconomic Health Disparities: A Tale of Two Asian Countries

**DOI:** 10.3390/ijerph18020717

**Published:** 2021-01-15

**Authors:** Cecilia Cheng, Fanny Cheng, Saloni Atal, Sarlito Sarwono

**Affiliations:** 1 Social and Health Psychology Laboratory, Department of Psychology, The University of Hong Kong, Pokfulam, Hong Kong, China; shpsylab@hku.hk; 2 Department of Psychology, Cambridge University, Downing St., Cambridge CB2 3EB, UK; ssa46@cam.ac.uk; 3 Faculty of Psychology, Universitas Indonesia, Kota Depok, Jawa Barat 16424, Indonesia

**Keywords:** coping, disparity, health, flexibility, social capital, socioeconomic status

## Abstract

A wealth of past studies documented that individuals of lower socioeconomic status (SES) are more susceptible to both acute and chronic life stress than those of higher SES, but some recent evidence documents that not all individuals from the lower SES group experience immense stress. The present study was grounded in theories of coping and psychological adjustment, and a dual process model was formulated to address some resolved issues regarding socioeconomic disparities in health. For a robust test of the proposed dual process model, data were collected from two Asian countries—Hong Kong and Indonesia—with different socioeconomic heritage and conditions. Consistent with the predictions of our model, the present findings revealed that coping flexibility was a psychological mechanism underlying the positive association between social capital and health for the lower SES group, whereas active coping was a psychological mechanism underlying this positive association for the higher SES group. These patterns of results were largely replicable in both Asian samples, providing robust empirical support for the proposed dual process model.

## 1. Introduction

Socioeconomic disparities in health have long been as a major issue of concern in many societies (e.g., [1,2]). Compared with individuals higher in socioeconomic status (SES), those lower in SES are 2.5 times more likely to be hospitalized and 3.5 times more likely to suffer from disability due to diseases [3,4]. However, some scholars argued that not all individuals of low SES would experience health problems, and psychological factors may influence the link between socioeconomic circumstance and health outcomes (e.g., [5,6]). In light of recent findings that coping flexibility could foster better health-related quality of life among individuals lower (vs. higher) in SES [7], it is necessary to refine the postulation of classic theories with SES as a risk factor of health by considering the influence of coping in stressful encounters.

**Figure 1:**
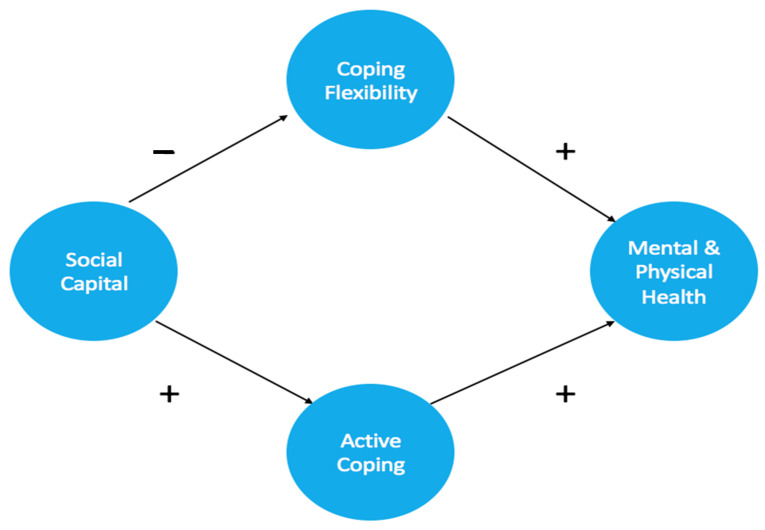
Conceptual framework of the proposed dual process model.

The present study extends the literature by proposing coping as a major psychological mechanism that explicates individual differences in health outcomes among distinct SES groups. Grounded in the transactional theory of coping [8], we formulate a dual process model to address the unresolved but important issues regarding socioeconomic disparities in health. Figure 1 depicts the conceptual framework of our proposed model.

### 1.1. Active and Flexible Coping as Underlying Psychological Mechanisms

Our proposed dual process model puts forward active coping and coping flexibility as two pathways that explicate the associations between SES and two health conditions. Coping refers to the thoughts and behaviors involved in managing the demands of stressful situations [9]. Earlier studies classified coping goals into two broad types: problem-focused versus emotion-focused. Problem-focused coping targets at direct management of stressors (e.g., problem solving) while emotion-focused coping targets at regulating thoughts and behaviors invoked by stressors (e.g., [10,11]).

Active (or approach) coping is a major coping style that refers to the predominant use of problem-focused coping in handling a myriad of stressors. When facing health-related issues, individuals adopting active coping tend to seek professional help, informational support, or both with an intention to resolve the issues. Previous studies have documented positive associations of active coping with both mental and physical health. For instance, a recent study indicates that patients treated with hemodialysis who use problem-focused coping more frequently are characterized by longer survival, improvement in physical functioning, and better mental health, compared with their counterparts who use this strategy less frequently [12]. Another study with self-identified lesbian, gay and bisexual community members reported inverse associations of active coping with both depression and anxiety [13].

The transactional theory of coping [8] postulates coping as a dynamic process such that the deployment of problem-focused or emotion-focused coping strategies depends primarily on the effectiveness of a particular strategy in managing the specific stress event at hand (i.e., flexible coping). If a stressor is perceived to be controllable, problem-focused coping should be more situationally appropriate than emotion-focused coping. In contrast, if another stressor is perceived to be uncontrollable, emotion-focused coping should be more situationally appropriate than problem-focused coping. To tackle health-related issues, individuals characterized by flexible coping tend to evaluate whether those issues are controllable or not, and then deploy a coping strategy that is the most effective in managing the issues. Studies have shown the beneficial role of flexible coping in psychological adjustment and in improving physical health. For example, inverse associations of coping flexibility with depression, anxiety, and distress have been similarly obtained for both university students and working adults [14]. Patients with psychosomatic symptoms who deploy flexible coping are found to report less somatic symptoms and higher physical quality of life [15,16]. Taken these two bodies of findings into consideration, we predict that both active and flexible coping will be positively associated with mental and physical health, but their benefits tend to vary among individuals with distinct demographic characteristics.

### 1.2. Individual Differences in Social Capital and Coping

In our proposed dual process model, individuals from different SES groups are predicted to deploy distinct types of coping for stress management due to variations in resource accrual. Such a notion stems from classic theories of social class (e.g., [17]), which postulate that individuals higher (vs. lower) in SES tend to deploy active coping due to the availability of greater socioeconomic resources available for direct handling of the stressors. The notion is also consistent with the findings yielded in some previous studies (e.g., [18,19]). In light of these theories and empirical evidence, we predict the same phenomenon to be observed in individuals equipped with higher levels of social capital such that they are more prone to use active coping and more apt to mobilize their social capital to handle stressors than those with lower levels of social capital.

In contrast, we predict that individuals lower (vs. higher) in SES tend to deploy flexible coping in stressful situations with an intention to optimize the benefits of the limited social capital available and to foster psychological adjustment. A meta-analytic review lends support to this prediction by revealing a stronger link between coping flexibility and psychological adjustment among individuals of lower SES compared with those of medium or higher SES [20]. In addition, a recent study on an Indian sample documented that among the low SES group, the respondents with higher coping flexibility reported better health than those with lower coping flexibility [7]. Accordingly, we predict different patterns regarding the association between social capital and coping: a positive association between social capital and active coping, as well as an inverse association between social capital and flexible coping.

### 1.3. Aims and Context of the Present Study

The present study aims to test the proposed dual process model in the context of Asian cultures. Asia is deemed an ideal context of studying both active and flexible coping because a meta-analysis indicates the beneficial role of coping flexibility in health enhancement as more prominent in collectivistic than individualistic societies [20]. Over recent decades, Asian countries differ vastly in policies and practices to attain socioeconomic growth. It is essential to test the hypotheses among individuals from countries at diverse stages of socioeconomic development.

**Figure 2:**
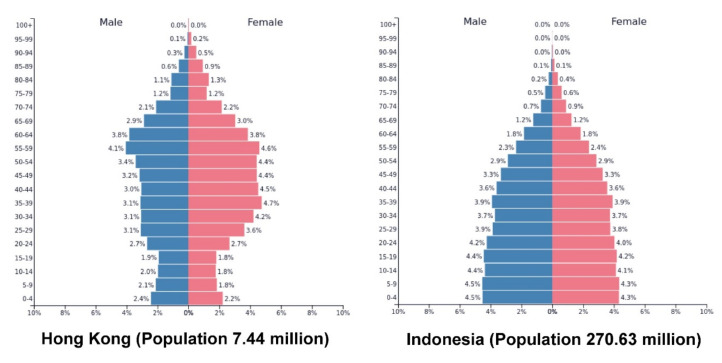
Population pyramids showing the distribution of sex and age groups in the population of Hong Kong and Indonesia in 2020 (PopulationPyramid.net, 2020).

For a robust test of our proposed model, the study was conducted in Hong Kong and Indonesia that differ in several major aspects. One prominent difference is that the two countries are characterized by dissimilar demographic structures. As shown in Figure 2, young people currently constitute the largest proportion in the population pyramid of Indonesia, while older adults currently constitute the largest proportion in the population pyramid of Hong Kong. Another prominent difference is that the proportion of residents who have completed high school education is higher for Hong Kong (>60%) compared with Indonesia (<35%; [21]).

In terms of socioeconomic development, Hong Kong has experienced accelerated socioeconomic growth since the 1970s, whereas Indonesia experienced such growth in the late 20th century [22]. In 2019, the gross domestic product (GDP) per capita of Hong Kong (US$48,756) was tenfold more than that of Indonesia (US$4136; [21]]. However, Hong Kong has a much steeper wealth gap (currently ranked 9 on the World Gini index), whereas the problem of wealth distribution is less severe for Indonesia (currently ranked 83 on the World Gini index; [23]).

Apart from differences in the pace of socioeconomic growth and wealth distribution, the two countries also vary vastly in their focus of and expenditure on an array of welfare regimes. Specifically, Hong Kong has higher public expenditure on both healthcare and education than Indonesia, whereas Indonesia has higher public expenditure on social security and welfare in general than Hong Kong [24]. Taken together, testing a psychological model in these two countries with diverse demographic structures, socioeconomic heritage, and welfare regimes thus provide a more robust analysis that enhances the generalizability of new findings yielded from this study.

## 2. Methods

### 2.1. Study Design

The present study adopted structured interviews because the questionnaire method was inappropriate for certain participants in a community sample with heterogeneous demographic characteristics (e.g., older adults, individuals with low education levels). The quota sampling method was adopted. Specifically, we used a balanced data design with a uniform sample quota across three SES subgroups: low, middle, and high. In addition, this study adopted a sex- and age-matched design because these two demographic variables were found to influence coping and psychological adjustment. Effort was made to maintain an equal sex and age-group ratio for all three SES subgroups.

### 2.2. Participants and Procedures

A priori power analysis for a 2 (Country) ×3 (SES Subgroup) design showed that a minimum of 276 participants allow for the detection of a medium effect size with 80% power at an alpha level of 0.05 [25]. Hence, we recruited a slightly larger total sample of 300 native adults, with 50 in each condition. In each country, the participants were recruited through quota sampling in three SES categories: low, middle, and high. Potential participants were initially screened through an objective, multidimensional measure, namely the revised Kuppuswamy Scale [26], which measured SES in terms of education, occupation, and income. Eligible participants were then invited to attend an interview session until each of the quotas was filled. Although it is ideal to recruit an equal number of male and female participants, it was far more difficult to recruit male participants from the low SES cluster than those from the medium and the high SES clusters. To maintain an equal sex- and age-group ratio across the three SES groups, we recruited the participants from the low SES group first, and then matched the sex and age distributions of those from the other two SES groups in the recruitment process.

For both countries, each SES subgroup comprised 14 male and 36 female working adults. The median age was 37 years, with 30% between 19 and 30 years old, 42% between 31 and 45 years old, and 28% between 46 and 59 years old. These three age groups were evenly distributed in each SES subgroup for both countries.

The study protocol received ethical approval from the Human Research Ethics Committee at the University of Hong Kong before the two arms of this project began. The participants were interviewed in a cubicle in a research institute or community center from which they were recruited. They were asked to give informed consent at the outset of the interview. After the interview had been completed, all of the participants were debriefed and thanked for their participation.

### 2.3. Measures

The Personal Social Capital Scale [27] was employed to measure levels of personally owned social capital. Respondents rated this 10-item scale on a 5-point scale, ranging from 1 (strongly agree) to 5 (strongly disagree). Higher scores indicate greater strength and size of social networks. This scale displayed good psychometric properties in Chinese samples [28].

The deployment of both coping flexibility strategies and active (vs. avoidant) coping strategies was assessed by the Coping Flexibility Interview Schedule [15], which comprised two sections. In the first section, respondents were instructed to describe both controllable and uncontrollable stressors recently experienced. In the second section, they recalled all of the coping strategies deployed to handle each of their reported stressors. Then they stated the primary goal of deploying each of these strategies by indicating whether they used it for directly confronting or tackling a particular stressor (i.e., active coping), or they simply did nothing or tried not to face the stressor (i.e., avoidant coping).

Upon completion of the interview, a research assistant scored the answers given by each respondent using a previously validated coding scheme. To obtain a coping flexibility score, the research assistant assigned a score of 1 to the use of active coping in a controllable stressor or avoidant coping in an uncontrollable stressor. A score of 0 was assigned to all other answers. As respondents varied vastly in the number of stressors and coping strategies reported, the research assistant aggregated the scores and then divided the scores by the total amount of coping strategies employed.

To obtain an active (vs. avoidant) coping score, the research assistant then counted the number of active and avoidant coping strategies, respectively; and divided the number of active coping strategies by the total number of coping strategies deployed. Both of these coping scores ranged from 0 to 1. The Coping Flexibility Interview Schedule had been validated in Chinese samples [10,15].

Self-ratings of both physical and mental health were tapped by the Short Form-8 Health Survey. This measure consisted of four items assessing physical health and four assessing mental health. For each of these health domains, the scores of four individual items were aggregated to form a composite score, which was then standardized to a 100-point index [29]. Higher scores indicated better perceived health in the particular domain. The Short Form-8 Health Survey was found to be reliable and valid in both Chinese and Indonesian samples [30,31].

### 2.4. Statistical Analysis

Multivariate analysis of variance (MANOVA) was performed to test the hypothesized group differences. If a statistically significant difference was found for a variable with two levels (i.e., country, sex), post hoc independent samples *t*-test would be employed to further clarify the direction of differences. If a statistically significant difference was found for a variable more than two levels (i.e., SES), one-way analysis of variance (ANOVA) was employed that was followed by Bonferroni test, which compared each pair of subgroups for detecting statistically significant differences among the various subgroups.

Before conducting these statistical analyses, various assumption checks were conducted. Specifically, graphical boxplot was inspected to test the assumption of the absence of significant outliers in the data. The Shapiro-Wilk goodness-of-fit test was performed to test the assumption of normality, with non-significant results indicating normal distribution of data. The Levene’s test for equality of variances was employed to test the assumption of homogeneity of variance among subgroups of comparison, and non-significant results indicated that this assumption was not violated. If any assumption was violated, data transformation or non-parametric analyses would be needed.

The data were analyzed with statistical software of IBM-SPSS Statistics for Windows version 23.0 (Armonk, NY: IBM Corp.), JAMOVI version 1.2.2.0 [32], and SmartPLS version 3.0 [33]. The *p* values less than 0.05 were considered statistically significant in this study.

## 3. Results

### 3.1. Preliminary Analyses

The graphical boxplots identified no outliers in any of the study variables. The Shapiro-Wilk goodness-of-fit test showed that the various variables were normally distributed, *p*s > 0.05. The Levene’s tests for equality of variances further revealed homogeneity of variance among the subgroups of comparisons, *p*s > 0.19. Taken together, the preliminary analyses showed that none of the assumptions were violated.

### 3.2. Descriptive Statistics

Table 1 presents the means and standard deviations of the five study variables, namely social capital, coping flexibility, active (vs. avoidant) coping, physical health, and mental health.

### 3.3. Country and SES Differences

The results of MANOVA revealed that the overall differences in the levels of the five study variables between the Hong Kong and the Indonesian samples were statistically significant [*F* (5, 290) = 4.424, *p* = 0.001, partial eta squared = 0.071, Wilks’ Lambda = 0.929]. However, post hoc independent samples *t*-test revealed marginal country differences in levels of social capital and mental health [social capital: *t*(298) = 1.797, *p* = 0.073 and mental health: *t*(298) = −1.721, *p* = 0.086], with the participants from Hong Kong reporting higher levels of social capital but lower levels of mental health than their counterparts from Indonesia.

The MANOVA results also showed that the overall differences in the levels of study variables among the three SES subgroups were statistically significant [*F* (10, 580) = 37.609, *p* < 0.001, partial eta squared = 0.393, Wilks’ Lambda = 0.368]. Table 2 summarizes the results of the Bonferroni tests that compared each pair of SES subgroups for detecting statistically significant differences in levels of social capital among the three SES subgroups, and statistically significant difference of coping flexibility, active coping, and physical health composite scores at varying SES levels. As shown in this table, the participants of the medium SES subgroup, including both from Hong Kong and Indonesia, had the highest coping flexibility scores (M = 0.54) and those of the high SES subgroup had the lowest coping flexibility scores (M = 0.47). For active coping, the participants of the high SES subgroup scored the highest (M = 0.56), followed by the medium (M = 0.48) and low (M = 0.47) SES subgroups. The same pattern was observed in social capital scores (Ms = 25.14, 21.88, and 12.71 for high, medium, and low SES subgroups, respectively).

For physical and mental health, the participants of the high SES subgroup scored the highest in physical health (M = 51.70), closely followed by that of the medium SES subgroup (M = 50.32), while the high (M = 53.17) and the medium (M = 53.14) SES subgroups scored almost identical in mental health.

Although the overall main effects of country and SES were statistically significant, the Country × SES interaction effect was not statistically significant [*F* (10, 580) = 1.382, *p* = 0.185, partial eta squared = 0.023, Wilks’ Lambda = 0.954].

### 3.4. Path Analysis

**Figure 3:**
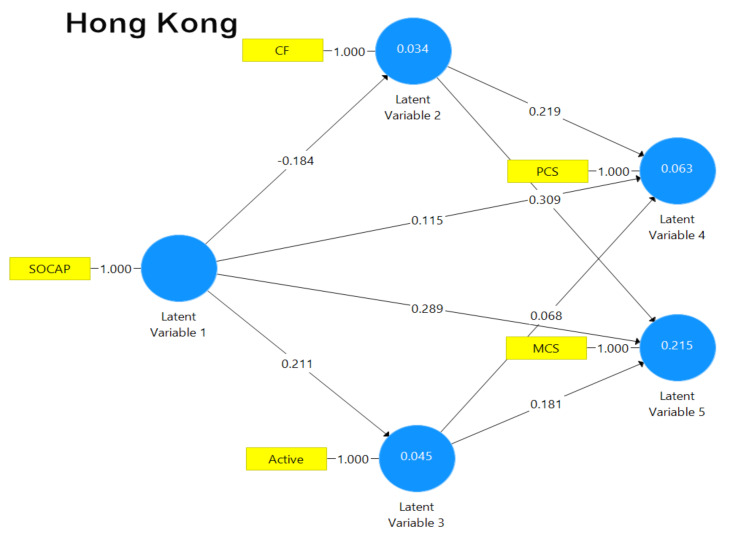
Diagram summarizing the path analytic findings of the Hong Kong sample. [Note. Active = active coping; CF = coping flexibility; MCS = mental health composite score; PCS = physical health composite score; SOCAP = social capital].

**Figure 4:**
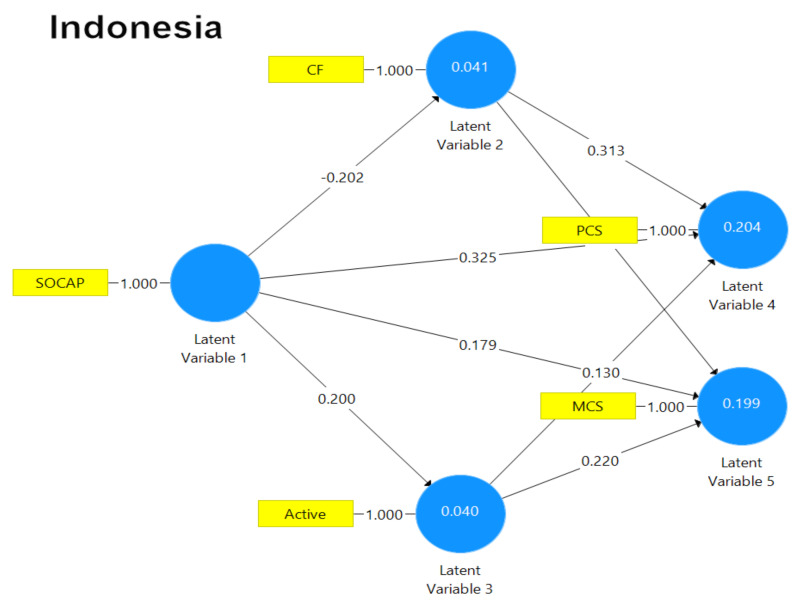
Diagram summarizing the path analytic findings of the Indonesian sample. [Note. Active = active coping; CF = coping flexibility; MCS = mental health composite score; PCS = physical health composite score; SOCAP = social capital].

The results of path analysis for the Hong Kong and the Indonesia samples are graphically depicted in Figure 3 and Figure 4, respectively. As shown in Figure 3, all the paths (except the two from social capital to physical health and from active coping to physical health, respectively) were statistically significant. As predicted, the path coefficients indicated that the associations between the variables were mostly positive while an inverse association was detected for the path between social capital and coping flexibility. Overall, the path model for the Hong Kong sample accounted for 26% of the variance.

Figure 4 shows that for the Indonesian sample, all the paths (except the two from social capital to mental health and the one from active coping to physical health, respectively) were statistically significant. As predicted, the path coefficients indicated that the associations between the variables were mostly positive and similar to the Hong Kong sample (see Figure 3), with an inverse association observed for the path from social capital to coping flexibility. Overall, the path model for the Indonesia sample explained 36% of the variance.

## 4. Discussion

The present study formulated and tested a dual process model in which active coping and flexible coping are proposed as two pathways to explain the associations between SES and two indicators of well-being, namely mental and physical health. More importantly, SES is often studied from economic and financial perspectives, using income, education, and occupation as indicators [34,35]. The present study expanded the scope of SES from a personal to an interpersonal perspective, specifically, in terms of the levels of social capital accrued, and examined how Asians with varying levels of social capital deploy such resources and coping strategies for handling stress, which in turn facilitates psychological adjustment.

### 4.1. Dual Process Model

The predictions of the proposed dual process model are found to be applicable to both developed (Hong Kong) and developing (Indonesia) Asian countries. Considerable individual differences in strategy deployment are found in each country, yet the underlying mechanisms are largely replicable in both. These replicable findings thus provide robust support showing that social and personal resources assessed at the individual level play an influential role on health issues.

The new findings contribute to the literature in three major ways. First, the present study indicates that in stressful situations, individuals with more (vs. less) social capital are more prone to use active coping as their primary strategy, whereas those with less (vs. more) social capital are more prone to use flexible coping as their primary strategy. These findings demonstrate that individuals with more (vs. less) social capital tend to use active coping because more socioeconomic resources are available to them for stress management, whereas individuals with less (vs. more) social capital tend to use flexible coping with the intention to optimize the benefits of their limited resources [20].

Second, the use of flexible coping has significant mental and physical health benefits among Asians, who are characterized by a dialectical thinking style [36]. The dynamic process of flexible coping promotes adaptiveness to stressful events such that individuals adopting flexible coping may deploy problem-focused or emotion-focused coping to manage the specific demands of distinct stressful events deemed situationally appropriate. These findings are corroborated by those yielded from Asian studies, which indicate that coping flexibility is inversely associated with levels of depressive symptoms but positively associated with both psychological and physical well-being [14,37,38].

Third, also consistent with the literature (e.g., [12,13]), the use of active coping has mental health benefits. In stressful situations, individuals adopting active coping tend to make efforts (e.g., gather information and planning) and focus on managing the problems that elicit psychological distress. Hence, they may gain a better sense of self-efficacy, mastery, and control of the stressors [39]. Previous studies have also indicated that active coping is positively associated with hope and optimism [40] but inversely associated with depression [41].

### 4.2. Mixed Findings on the Social Capital-Health Associations

Two diverging associations of social capital and health observed between the two countries deserve further scrutiny. First, the association between social capital and physical health was significant in the Indonesian sample rather than the Hong Kong sample. Second, there was a reverse pattern of findings with respect to mental health revealing an insignificant association between social capital and mental health in the Indonesian sample, but such an association was significant in the Hong Kong sample. A possible explanation for these unexpected mixed findings is the differential effects of bridging versus bonding social capital on health. This notion stems from a study conducted in Mainland China that has similarly revealed empirical inconsistencies between social capital and self-rated health status [42], with the pattern of such associations tended to vary by urban versus rural communities. Specifically, Chinese adults residing in rural areas tend to report a positive association between bonding social capital and health status, but this association is largely absent among those residing in urban areas. These complex results highlight the importance to differentiate between bonding versus bridging social capital as well as between urban versus rural residents.

The effects of social capital on mental and physical health may also differ from one SES group to another. While individuals with ample resources may find support rendered by their social network members to be helpful, those with scant resources may consider such support a burden due to an obligation to reciprocate, which may be the case due to their limited resources [43]. Although social support has been widely viewed to bolster physical and mental well-being [44], considerable cross-cultural differences in social support seeking have been identified. Both Asians and Asian Americans are found to be less likely to seek social support due to relational concerns (e.g., failure to reciprocate, assistance rendered with strings attached) than their Western counterparts [45,46]. These cultural differences thus reflect that social support may be detrimental to health among Asians under certain circumstances, such as those with scant social resources that hinder their likelihood of repaying the support provider.

### 4.3. Non-Significant Active Coping-Physical Health Associations

Differing somewhat from our hypothesis, the present results fail to reveal a significant association between active coping and physical health in both Hong Kong and Indonesian samples. This discrepancy may be due to cultural differences in thinking style. Coping research has consistently identified the stress-buffering role of active coping in Western countries (e.g., [47,48]), and the adoption of this direct action-oriented strategy matches the linear thinking style that is prominent among Westerners [49].

Active coping, however, may be deemed socially desirable for Asians who value interpersonal harmony [50,51], primarily because this coping strategy often involves direct confrontation that can jeopardize the quality of social relations and disrupt interpersonal harmony. Excessive use of active coping has been found to be associated with heightened anxiety levels among Chinese patients with psychosomatic symptoms [15]. Hence, flexible coping is especially beneficial for Asians (e.g., [37,38]), primarily because the deployment of this holistic type of coping takes the context into consideration that matches the dialectic thinking style prominent among Asians [36].

### 4.4. Research and Practical Implications

The present novel findings have implications for researchers. In the literature, most cross-cultural comparisons among countries have been conducted based on the individualism-collectivism framework [52,53]. Multinational studies espousing this approach tend to investigate cultural differences between individualistic and collectivistic countries [54], assuming considerable uniformity in cultural values and norms within each broad cluster of countries. However, the present study is among the few to make comparisons between two collectivistic countries with diverse demographic and socioeconomic characteristics. Although the findings yielded from the two countries are largely consistent, some notable differences in the pattern of findings are also unveiled. These new findings shed light on the value of espousing a nuanced approach to provide fine-grained analysis within the wide spectrum of countries along the cultural dimension of individualism-collectivism.

Our new findings also have implications for healthcare professionals in the design of intervention programs. In Asia, many existing stress management programs are modeled from those developed by Western scholars, and the Asian programs focus primarily on building coping and interpersonal skills that directly tackle life problems. However, the present findings reveal that active coping may not be optimal for clients from Hong Kong and Indonesia, who are characterized by a dialectical thinking style [36]. Rather, flexible coping is found to have greater mental health benefits than active coping for Asians. Healthcare professionals may incorporate modules of coping flexibility intervention that espouses a more holistic approach to stress management into program design.

For welfare workers, a common problem is that their beneficiaries may not necessarily have the resourcefulness to use these resources to their full potential or in the ways intended, thereby leading to resource waste or mismatch. To tackle this problem, the coping flexibility intervention may be tailored to clients with scarce social resources, strengthening their coping ability by broadening their coping repertoires, and more importantly, to foster discriminative thinking that facilitates more efficient and effective use of their limited socioeconomic resources available [55]. However, the overarching goal and focus of the coping flexibility intervention may differ for clients with greater social resources. As these clients have already been endowed with ample resources, it is essential for them to acquire cognitive-behavioral skills to sustain self-reliance in coping with life challenges and resourcefulness. In light of the present findings, it is imperative for both healthcare professionals and welfare workers to be attentive to the SES levels of their clients, and tailor the intervention design to match the specific needs and psychological characteristics of clients from diverse SES backgrounds.

### 4.5. Limitations and Research Directions

Several limitations of our study should be mentioned. First, the participants of our study are from Hong Kong and Indonesia. Although both are Asian countries, they differ considerably in certain major aspects that may confound the present findings. For instance, the dissimilar population structures of Hong Kong and Indonesia suggest that residents from the two countries may encounter distinct societal problems, and thus the sources of life stress may vary among the demographic groups. Specifically, Figure 2 shows that Indonesia has an expansive population pyramid reflecting a young and growing population, and unemployment among young people may constitute the core societal problem in this country, especially in times of recession [56]. In contrast, Hong Kong has a constrictive population pyramid reflecting an aging and shrinking population, and aging problems are prevalent in the country such as inadequate government-subsidized residential care services [57]. Moreover, the two countries differ in the extent of religious diversity. The religion of Hong Kong has a multi-faith diversity comprising both traditional Chinese (e.g., Buddhism, Taoism) and foreign (e.g., Christianity, Muslim) religions [58], and about half of its residents does not have a religion [59]. In contrast, Islam is the dominant religion in Indonesia [59]. As individuals with different religions have been found to deploy distinct types of coping (e.g., [60,61]), it is possible that the present results may be confounded by the religion held by the respondents. Future studies should assess all of these potential confounding factors to scrutinize their potential influence on stress and strategy deployment when making between-country comparisons.

Second, researchers should explore whether our findings, derived from Hong Kong and Indonesia, are generalizable to populations of other Asian countries and cultural regions. For example, in a study examining the association between social capital and mental health of women from Ethiopia, India, Peru, and Vietnam [62], the findings indicate that social capital is cultural-specific depending on the civic structure and the norms of participation of the relevant groups. Investigation of the proposed dual process model in other Asian countries and cultural regions is recommended to evaluate their extent of applicability across nations in diverse world regions.

Third, we have expended effort in recruiting a heterogeneous sample of community adults with a broad age range and representation of the entire SES spectrum to enhance the generalizability of our findings. Despite such methodological advantages, the potential problem of sample heterogeneity should be noted. As most psychological measures are developed from middle-class samples residing in urban areas, their items may be interpreted in a distinct manner by respondents from dissimilar demographic and socioeconomic backgrounds, such as older adults and rural residents (e.g., [63,64]). For instance, health is often self-assessed by respondents yet its meaning may vary vastly among individuals. A previous study has found that among individuals who self-reported fair or poor health, those who had higher income or education are at greater risks of mortality than those who received less income or education [65]. Researchers should be sensitive to such potential methodological issues when conducting studies among heterogeneous community samples.

For research directions, lower-income countries are noted to be underrepresented in the literature [20]. As the present findings have shown some differences in psychological adjustment between participants from developed countries and those from developing countries, we advocate more investigations to be conducted on flexible coping and health with samples from lower- and lower-middle-income countries. Additional demographic information (e.g., ethnicity, religion) should be assessed such that their potential confounding effects can be detected and controlled for. Lastly, objective measures, ratings by a third party (e.g., physician, spouse) are recommended to be administered with heterogeneous community samples. If self-report measures are to be used, additional effort should be made to evaluate whether the items are equally applicable across diverse SES groups and geographic areas.

### 4.6. Conclusions

The present study has expanded the scope of SES from personal to interpersonal resources, namely social capital. Furthermore, the study provides considerable support for the proposed dual process model that in stressful situations, individuals with more social capital tend to use active coping while those with less social capital tend to use flexible coping. In light of our findings, the classic theories that SES is a risk factor to health may be refined by taking individuals’ strategy deployment into consideration.

## Figures and Tables

**Table 1:** Descriptive statistics by country and SES subgroup.

	Country	SES Subgroup	Mean	SD	*n*
Social	Hong Kong	Low SES	13.10	3.234	50
Capital		Med SES	22.68	5.133	50
		High SES	26.08	4.458	50
	Indonesia	Low SES	12.32	3.254	50
		Med SES	21.08	4.873	50
		High SES	24.20	4.891	50
Coping	Hong Kong	Low SES	0.5620	0.20894	50
Flexibility		Med SES	0.5245	0.24549	50
		High SES	0.4635	0.21790	50
	Indonesia	Low SES	0.4755	0.24972	50
		Med SES	0.5555	0.20586	50
		High SES	0.4760	0.18280	50
Active	Hong Kong	Low SES	0.4430	0.29735	50
Coping		Med SES	0.4819	0.23639	50
		High SES	0.5720	0.32288	50
	Indonesia	Low SES	0.4870	0.26732	50
		Med SES	0.4865	0.28018	50
		High SES	0.5545	0.24675	50
Physical	Hong Kong	Low SES	50.76	12.640	50
Health		Med SES	49.78	11.134	50
		High SES	50.96	11.012	50
	Indonesia	Low SES	45.10	12.541	50
		Med SES	50.86	12.103	50
		High SES	52.44	11.262	50
Mental	Hong Kong	Low SES	49.68	11.991	50
Health		Med SES	52.26	11.108	50
		High SES	51.94	12.060	50
	Indonesia	Low SES	52.68	13.175	50
		Med SES	54.02	12.804	50
		High SES	54.40	11.681	50

Note. SES = socioeconomic status.

**Table 2:** Significant results of post hoc Bonferroni test on SES subgroup pairs.

Variable	SES Subgroup Pairs	*p*
Social Capital	Low SES (M = 12.71) vs. Medium SES (M = 21.88)	*p* < 0.001
	Medium SES (M = 21.88) vs. High SES (M = 25.14)	*p* < 0.001
	Low SES (M = 12.71) vs. High SES (M = 25.14)	*p* < 0.001
Coping Flexibility	Medium SES (M = 0.54) vs. High SES (M = 0.47)	*p* = 0.074
Active Coping	Low SES (M = 0.47) vs. High SES (M = 0.56)	*p* = 0.037
Physical Health	Low SES (M = 0.47.93) vs. High SES (M = 51.70)	*p* = 0.076

Note. SES = socioeconomic status.

## Data Availability

The data presented in this study are available on request from the corresponding author. The data are not publicly available due to the adherence to the conditions stated in the research protocol submitted for human research ethical approval.
